# Supplementary material for: Synergistic Anticancer Effect of Glycolysis and Histone Deacetylases Inhibitors in a Glioblastoma Model
Source: Biomedicines. 2021 Nov 23;9(12):1749. doi: 10.3390/biomedicines9121749 (PMC8698815; doi:10.3390/biomedicines9121749)
Supplement: Supplementary file 1 [file biomedicines-09-01749-s001.zip › biomedicines-1401560-supplementary.pdf]

biomedicines

MDPI

Synergistic Anticancer Effect of Glycolysis and Histone Deacetylases Inhibitors in a Glioblastoma Model

Beata Pająk [1,*], Ewelina Siwiak-Niedbalska [1], Anna Jaśkiewicz [1], Maja Sołtyka [1], Rafał Zieliński [2], Tomasz Domoradzki [1], Izabela Fokt [2], Stanisław Skóra [2] and Waldemar Priebe [2,*]

[1] Independent Laboratory of Molecular Biology and Genetics, Kaczkowski Military Institute of Hygiene and Epidemiology, Kozielska 4, 01-163 Warsaw, Poland; ewelinasiwiak1@op.pl (E.S.-N.); ancpatrin@gmail.com (A.J.); maja.soltyka@gmail.com (M.S.); domoradzki.tomasz@gmail.com (T.D.)

[2] Department of Experimental Therapeutics, The University of Texas MD Anderson Cancer Center, 1901 East Rd., Houston, TX 77054, USA; RJZielinski@mdandreson.org (R.Z.), ifokt@mdanderson.org (I.F.), sskora@maandreson.org (S.S.)

* Correspondence: bepaj@wp.pl (B.P.); wpriebe@mac.com (W.P.)

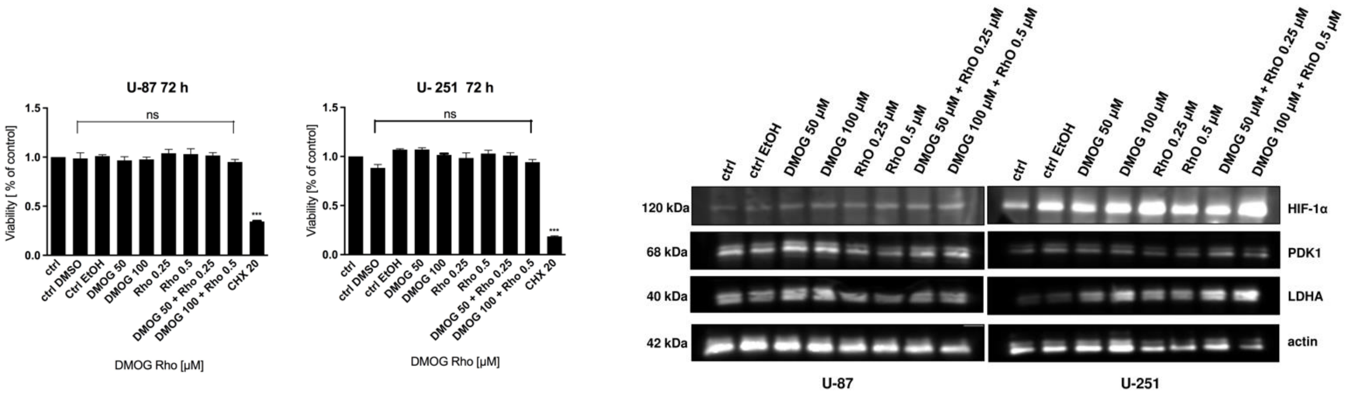

Figure S1. Hypoxia-like model. A) Viability of U-87 and U-251 cells treated with DMOG [50 and 100 uM] or/and rhodamine (Rho [0.25 and 0.5 uM]. As a positive control of cytotoxicity CHX was used [20 uM]. Significant differences between the treatment means and control value are indicated by * P < 0.05, ** P < 0.01 and *** P < 0.001. B) Representative Western blot analysis showing the expression of HIF-1α and its downstream proteins – PDK1 and LDHA after DMOG [50 and 100 uM] or/and rhodamine (Rho) [0.25 and 0.5 uM] treatment. Actin level was used as a loading control. Each experiment was replicated at least 3 time with similar results.

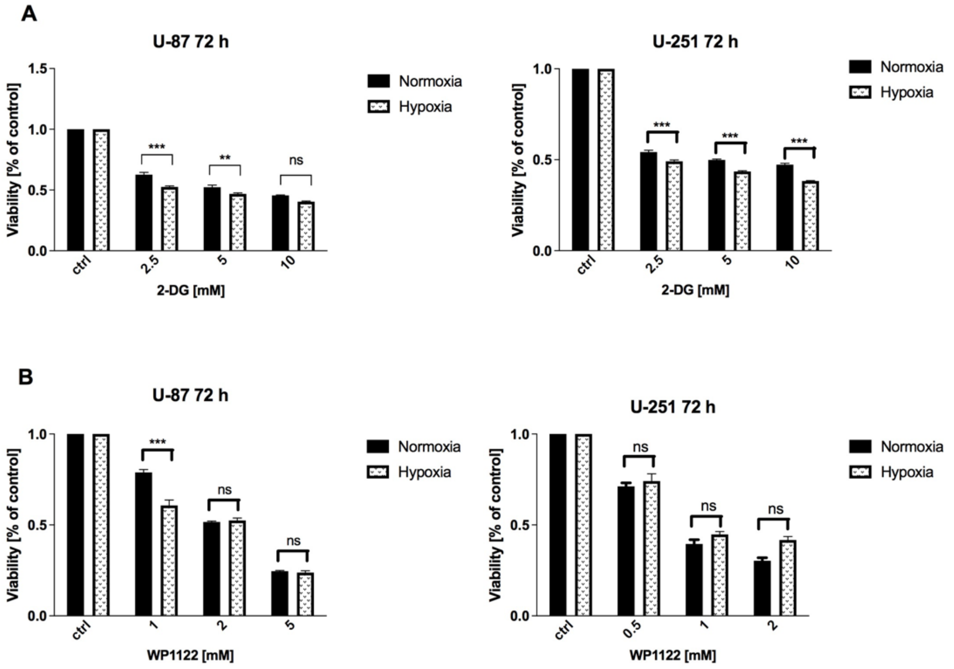

Figure S2. MTS assay showing viability of U-87 and U-251 cells after 72 h treatment with various concentrations of A) 2-DG [0.5-20 mM] and B) WP1122 [0.25-5 mM] in normoxia and hypoxia-like (DMOG + Rho) conditions. Significant differences between the treatment means and control value are indicated by * P < 0.05, ** P < 0.01 and *** P < 0.001.

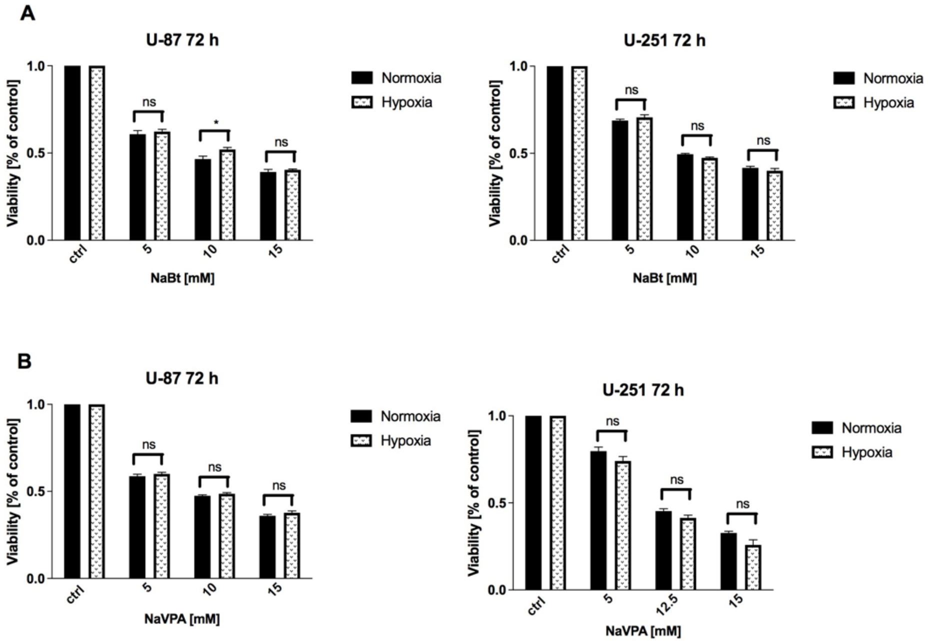

Figure S3. MTS assay showing viability of U-87 and U-251 cells after 72 h treatment with various concentrations of A) NaBt [5-15 mM] and B) NaVPA [5-15 mM] in normoxia and hypoxia-like (DMOG + Rho) conditions. Significant differences between the treatment means and control value are indicated by * P < 0.05, ** P < 0.01 and *** P < 0.001.

Table S1. Densitometric analysis of Western blot (Figure 11).

		U-87				
**Ratio OD protein/OD actin**	**Ctrl**	**Ctrl DMSO**	**2-DG**	**WP1122**	**NaBt**	**NaVPA**
**Bcl-2**	1.25	1.08	1.10	0.93	0.70	0.95
**Bad**	1.66	1.89	1.83	1.82	2.79	2.81
**Bax**	0.47	0.84	0.92	1.16	2.06	1.10
		**U-251**				
**Bcl-2**	1.05	1.00	1.48	1.10	0.79	0.73
**Bad**	1.13	1.19	1.34	1.29	0.75	1.30
**Bax**	0.89	0.93	0.64	0.61	1.64	1.07

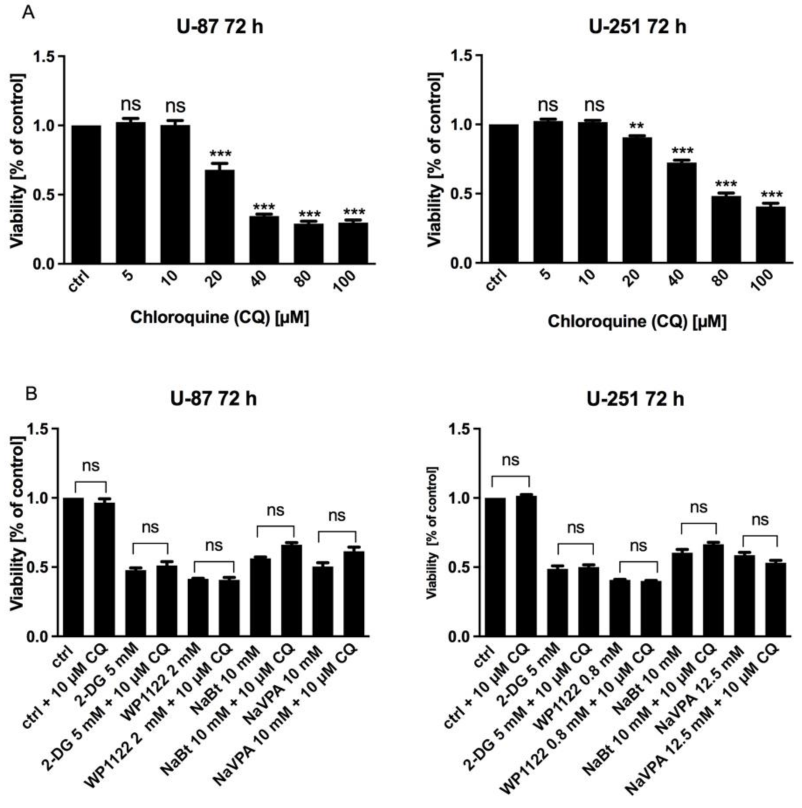

Figure S4. MTS assay showing viability of U-87 and U-251 cells after A) various concentrations of chloroquine (CQ) [5-100 uM], 72 h and B) combined treatment of CQ [10 uM] with IC50 concentration of 2-DG, WP1122, NaBt or NaVPA, 72 h. Significant differences between the treatment means and control value are indicated by * $P < 0.05$, ** $P < 0.01$ and *** $P < 0.001$.

Table S2. The summary of the drug-drug interaction assay with ChouTalalay method in U-87 cells. CI – combination index; Dm – mean effective concentration, IC50 value; m – determines the slope of the plot, the shape of the dose-response curve: m=1, m>1, m<1 represent a hyperbolic, sigmoidal and planar sigmoidal shape, respectively; r – Pearson's linear correlation coefficient between -1 and 1, the sign of the coefficient indicates the direction of the correlation (linear, negative or positive linear). Absolute value r reports the strength of the linear correlation. Red color indicates synergistic CI.

Summary table U-87						
**Drug**	**CI values at**					
	**ED50**	**ED75**	**ED90**	**Dm**	**m**	**r**
**2-DG**	N/A	N/A	N/A	4.31745	0.13395	0.98917
**(Not a combination)**						
**NaBt**	N/A	N/A	N/A	13.0742	0.45416	0.97161
**(Not a combination)**						
**1:10**	0.44244	3.54226	44.3666	4.05314	0.22201	0.98204
**1:5**	0.62521	1.38854	6.55277	4.58400	0.27644	0.96599
**1:2.5**	0.71368	0.48536	1.06754	3.98309	0.34180	0.95632
**1:1.25**	0.78643	0.11706	0.09421	3.32910	0.49583	0.94021
**2-DG**	N/A	N/A	N/A	6.31745	0.13534	0.98384
**(Not a combination)**						
**NaVPA**	N/A	N/A	N/A	13.57460	0.50279	0.94891
**(Not a combination)**						
**1:10**	0.34406	0.83120	2.58105	0.73077	0.33102	0.97429
**1:5**	0.42282	0.19718	0.14435	1.46837	0.55010	0.97345
**1:2.5**	0.37585	0.10679	0.06482	1.91260	0.55763	0.98629
**1:1.25**	0.30591	0.10704	0.12205	2.02862	0.47307	0.98908
**WP1122**	N/A	N/A	N/A	1.56980	1.98577	0.98320
**(Not a combination)**						
**NaBt**	N/A	N/A	N/A	10.4409	1.31452	0.94421
**(Not a combination)**						
**1:10**	0.58830	0.90167	1.40944	4.86748	0.98316	0.94361
**1:5**	0.49502	0.82458	1.39631	3.02069	0.95186	0.95346
**1:2.5**	0.59164	1.15578	2.28347	2.55599	0.86037	0.99112
**1:1.25**	0.57762	1.16627	2.37075	1.79611	0.85444	0.99059
**WP1122**	N/A	N/A	N/A	2.13384	1.43627	0.93241
**(Not a combination)**						
**NaVPA**	N/A	N/A	N/A	10.3325	0.46238	0.97068
**(Not a combination)**						
**1:10**	0.53352	3.40941	36.2360	4.08551	0.32364	0.98168
**1:5**	0.53974	0.69884	1.31619	3.39975	0.71103	0.98280

**1:2.5**	0.45132	0.72349	1.47499	2.22295	0.70652	0.93979
**1:1.25**	0.51130	0.76044	1.30008	1.95115	0.81912	0.97770

Table S3. The summary of the drug-drug interaction assay with ChouTalalay method in U-251 cells. CI – combination index; Dm – mean effective concentration, IC50 value; m – determines the slope of the plot, the shape of the dose-response curve: m=1, m>1, m<1 represent a hyperbolic, sigmoidal and planar sigmoidal shape, respectively; r – Pearson's linear correlation coefficient between -1 and 1, the sign of the coefficient indicates the direction of the correlation (linear, negative or positive linear). Absolute value r reports the strength of the linear correlation. Red color indicates synergistic CI.

Summary table U-251						
**Drug**	**CI values at**					
	**ED50**	**ED75**	**ED90**	**Dm**	**m**	**r**
**2-DG**	N/A	N/A	N/A	6.81406	0.17952	0.98637
			**(Not a combination)**			
**NaBt**	N/A	N/A	N/A	13.7716	0.52007	0.95971
			**(Not a combination)**			
**1:10**	0.49112	0.43252	0.48777	0.96401	0.76701	0.98505
**1:5**	0.47041	0.23307	0.17996	1.51037	1.04050	0.97901
**1:2.5**	0.43976	0.21561	0.22270	2.06991	0.81296	0.98290
**1:1.25**	0.40552	0.14957	0.17502	2.48856	0.73963	0.98285
**2-DG**	N/A	N/A	N/A	5.73824	0.25737	0.91640
			**(Not a combination)**			
**NaVPA**	N/A	N/A	N/A	11.8238	1.16732	0.99806
			**(Not a combination)**			
**1:10**	0.92220	0.55663	0.47719	9.5427	1.36784	0.97973
**1:5**	0.78921	0.34107	0.26731	7.1290	1.50646	0.99074
**1:2.5**	0.71946	0.21743	0.16601	4.41457	1.49708	0.86863
**1:1.25**	0.77658	0.17326	0.14310	3.91104	1.26125	0.93162
**WP1122**	N/A	N/A	N/A	2.30484	0.49654	0.96411
			**(Not a combination)**			
**NaBt**	N/A	N/A	N/A	10.0066	0.99120	0.98593
			**(Not a combination)**			
**1:10**	0.66720	1.37522	3.60890	6.27063	0.49949	0.97846
**1:5**	0.72596	0.48179	0.41053	5.67853	0.93625	0.97173
**1:2.5**	0.63307	0.45967	0.44565	3.69004	0.75866	0.96660
**1:1.25**	0.61516	0.33340	0.23728	2.67632	0.83308	0.98499
**WP1122**	N/A	N/A	N/A	2.90504	0.76728	0.89781
			**(Not a combination)**			
**NaVPA**	N/A	N/A	N/A	10.5045	1.42375	0.94475
			**(Not a combination)**			
**1:10**	0.98198	1.11483	1.37766	6.9841	0.97950	0.98270
**1:5**	0.96174	0.75293	0.65443	5.81234	1.27841	0.97260

**1:2.5**	0.48645	0.24450	0.13629	3.65242	2.09230	0.98054
**1:1.25**	0.69116	0.67945	0.72531	3.83795	0.85622	0.94445
